# Influence of Second-Hand Smoke and Prenatal Tobacco Smoke Exposure on Biomarkers, Genetics and Physiological Processes in Children—An Overview in Research Insights of the Last Few Years

**DOI:** 10.3390/ijerph17093212

**Published:** 2020-05-05

**Authors:** Markus Braun, Doris Klingelhöfer, Gerhard M. Oremek, David Quarcoo, David A. Groneberg

**Affiliations:** Institute of Occupational Medicine, Social Medicine and Environmental Medicine, Goethe University Frankfurt, D-60590 Frankfurt, Germany; klingelh@med.uni-frankfurt.de (D.K.); Oremek@gmx.net (G.M.O.); Quarcoo@med.uni-frankfurt.de (D.Q.); arbsozmed@uni-frankfurt.de (D.A.G.)

**Keywords:** environmental tobacco smoke, passive smoke, smoking in pregnancy, maternal tobacco smoke, asthma, allergy, atopy, immunity, wheezing, genetic predisposition

## Abstract

Children are commonly exposed to second-hand smoke (SHS) in the domestic environment or inside vehicles of smokers. Unfortunately, prenatal tobacco smoke (PTS) exposure is still common, too. SHS is hazardous to the health of smokers and non-smokers, but especially to that of children. SHS and PTS increase the risk for children to develop cancers and can trigger or worsen asthma and allergies, modulate the immune status, and is harmful to lung, heart and blood vessels. Smoking during pregnancy can cause pregnancy complications and poor birth outcomes as well as changes in the development of the foetus. Lately, some of the molecular and genetic mechanisms that cause adverse health effects in children have been identified. In this review, some of the current insights are discussed. In this regard, it has been found in children that SHS and PTS exposure is associated with changes in levels of enzymes, hormones, and expression of genes, micro RNAs, and proteins. PTS and SHS exposure are major elicitors of mechanisms of oxidative stress. Genetic predisposition can compound the health effects of PTS and SHS exposure. Epigenetic effects might influence in utero gene expression and disease susceptibility. Hence, the limitation of domestic and public exposure to SHS as well as PTS exposure has to be in the focus of policymakers and the public in order to save the health of children at an early age. Global substantial smoke-free policies, health communication campaigns, and behavioural interventions are useful and should be mandatory.

## 1. Introduction

Second-hand smoke (SHS) consists of mainstream smoke exhaled by a smoker and side-stream smoke from the smouldering tobacco product [1]. The terms “environmental tobacco smoke (ETS)” and “passive smoke” as synonyms for SHS are common but were also described as the sum of SHS and third-hand smoke [2]. Important sources of SHS exposure are the workplace, public places where smoking is allowed, smoker’s homes and vehicles. In particular, homes and vehicles are loci where children and pregnant women can be exposed to SHS [3]. Unfortunately, exposure to SHS in private homes and vehicles is still common [4,5].

SHS is linked to lots of health hazards. More than 7000 chemicals, including at least 70 carcinogenic substances, have been identified in SHS [3]. These carcinogens can cause several types of cancer (e.g., lung cancer, breast cancer, stomach cancer, cancers of the upper respiratory tract) [6,7]. Therefore, SHS increases the risk of children contracting lymphoma, leukaemia, liver cancer or brain tumours [8]. Additionally, SHS is harmful to heart and blood vessels, so it increases the risks of stroke and heart diseases in later life significantly [9]. SHS irritates the airways, can cause or worsen asthmatic diseases and allergies and is a major risk factor in chronic obstructive pulmonary disease (COPD). Tobacco smoke can also trigger lung infections and wheezing in children [10,11]. Exposed very young children are at increased risk of sudden infant death syndrome [8]. Not least, SHS seems to be linked with mental health effects [12]. Prenatal tobacco smoke (PTS) exposure can cause poor birth outcomes and pregnancy complications. PTS is linked with biochemical changes in the placenta, leading to alterations to the antioxidant system of the foetus, associated with several adverse health effects both prenatal and postnatal. PTS exposure can lead to pulmonary diseases, e.g., COPD, wheezing, asthma, kidney diseases as well as cardiovascular diseases in later life. Also, metabolic syndrome and obesity can be a consequence of PTS exposure [8,13].

**Figure 1:**
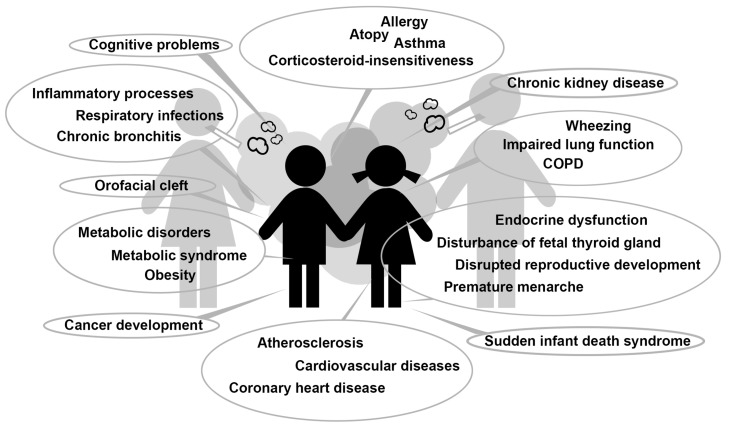
Adverse health effects in children induced by second-hand smoke and prenatal tobacco smoke exposure. COPD = chronic obstructive pulmonary disease.

Extensive data from epidemiological and experimental studies indicate that gene–environmental interaction during pregnancy and early life can induce permanent changes in physiological processes and disease predisposition by epigenetic mechanisms [14]. The early events during pregnancy and childhood play a key role in the development of the human body. In this vulnerable phase, exposure to tobacco smoke have been shown to have deleterious effects on the development process and may result in permanent damage [15]. The genetic predisposition can lead to substantially aggravated risk for diseases of children exposed to SHS [16,17,18,19]. It might be said that PTS is the first major environmental factor that can jeopardise the health of the unborn child (Figure 1) [13].

According to the World Health Organization (WHO), data from 2004 from 192 countries showed that 40% of children and about 33% of adult non-smokers were exposed to SHS worldwide [4,20]. In the United States, the exposition to SHS in non-smokers decreased from 52.5% from 1999 to 2000 to 25.3% from 2011 to 2012 (SHS exposed children aged 3–11 years: 40.6%) [21]. The European human biomonitoring pilot study (September 2011 to January 2012) reported SHS exposure in children aged 6–11 years from Portugal, Poland, and Romania [22]. Regarding the daily SHS exposure at home, the prevalence in Portugal was 15%, Poland 19.3%, and Romania 21.7%. Each year, more than 1.2 million premature deaths are caused by SHS, 65,000 of which are children. Nevertheless, only 22% of the world population is protected by comprehensive national smoke-free laws [23]. Lau and Celermajer [24] declared in 2014, 50 years after the US Surgeon General’s first report in 1964, that the protection of children from SHS is one of the great healthcare challenges.

This review explores a range of basic science research regarding biomarkers, physiological processes (Table 1) and genetics addressing PTS and SHS exposure. Additionally, a short overview will be given on changes in DNA methylation induced by PTS exposure. Molecular and genetic mechanisms underlying tobacco smoke-induced diseases are not completely understood, so further research will be necessary. However, this review aims to inform the policymakers and the public for a deeper understanding of the adverse health effects of tobacco smoke, especially in children.

## 2. Biomarkers

### 2.1. Matrix Metalloproteinase-9

The enzyme matrix metalloproteinase-9 (MMP-9), also known as gelatinase B, has the ability to regulate the activity of some soluble proteins and plays a role in the degradation of extracellular matrix components. It plays important parts in physiological and pathological processes [25], e.g., in the pathogenesis of allergy [26]. In this respect, levels of MMP-9 in the sputum of patients with chronic bronchitis or asthma were significantly higher than in a control group [27]. In a study from 2011, De et al. [26] investigated the effects of SHS on levels of MMP-9 in nasal secretions of 39 children aged between 7 and 16 years. The authors found that MMP-9 concentrations and activity were significantly higher in the probed nasal secretions of children exposed to SHS. They concluded that SHS might alter in the nasal mucosa the inflammatory reaction, similar to an allergic response. In a recent study, in children with cystic fibrosis (CF) exposed to SHS, it was found that the MMP-9 gene is overexpressed [28].

Yilmaz et al. [29] came to a different conclusion in an investigation of the influence of SHS exposure on nasal and serum inflammatory markers in 150 wheezing children (91 children exposed to lower levels of SHS, 24 children exposed to higher levels and 35 non-exposed children). Admittedly, the authors reported that SHS aggravated respiratory symptoms in exposed wheezing children significantly, albeit without significant influence on inflammatory markers like nasal MMP-9, tissue inhibitor of metalloproteinase-1 (TIMP-1), glutathione, interleukin-8 and 17 (IL-8, IL-17) and serum surfactant protein-D (SP-D).

### 2.2. Immune-Regulatory Cytokines

Interleukins (ILs), interferons (IFNs) and tumour necrosis factor-alpha (TNF-α) are immune-regulatory cytokines and therefore biomarkers of inflammatory processes [30]. These cytokines are associated, among others, with asthma, allergy and respiratory illness [10,31,32]. Chahal et al. [33] investigated the effect of PTS on levels of IL-1α, IL-6, IL-8 and IL-1 receptor antagonist in dried blood of new-borns. They ascertained an increase in IL-8 levels among neonates exposed to PTS. Additionally, a decrease in IL-1β, IL-4, IL-5, and IFN-γ was described in healthy 1–6-year-old SHS-exposed children [34]. In an investigation of children and adolescents aged 0–17 years, a positive association between high SHS exposure and IL-1β levels in saliva was found [35]. In saliva samples of five-year-old children exposed to SHS, the levels of IL-1β, IL-6 and TNF-α, but not IL-8, were elevated [36]. Airway IL-13 secretion was increased in SHS-exposed children [37].

The findings of these studies indicate that PTS and SHS exposure influences the levels of immune-regulatory cytokines with effects on inflammatory processes in children.

### 2.3. Cysteinyl Leukotrienes and Urinary Leukotriene E4

Cysteinyl leukotrienes (CysLTs) are key mediators and modulators in the pathogenesis of asthma [38]. Changes in CysLT expression and excretion can be detected by measuring urinary leukotriene E4 (uLTE4) levels [39]. In smokers, as well as in children exposed to tobacco smoke, leukotriene receptor antagonists (LTRAs) are more effective [40,41]. This shows the CysLT pathway plays an important role in mediating SHS-triggered asthma-related health effects [42]. In 2008, Kott et al. [43] ascertained a correlation between SHS exposure, increase in uLTE4 in exposed infants suffering from respiratory syncytial virus bronchiolitis and length of hospital stay.

In 2011, Rabinovitch et al. [42] proposed uLTE4 as the first biomarker to identify SHS-exposed children at risk of asthma exacerbation. As SHS is linked to increased asthma severity in affected children [44], the biomarker uLTE4 is important to estimate the predisposition of asthmatic children to severe exacerbations. In children with poor asthma control exposed to SHS, a trend for increasing uLTE4 concentrations was determined [45]. A study from 2013 approved the correlation between SHS and increasing uLTE4, but with the indication that the body mass index has still a more pronounced influence on its concentration [46]. One study found that in opposition to inhaled corticosteroids to treat asthma, oral treatment with the LTRA montelukast prevents an increase in uLTE4 level in children at low SHS exposure, but with no effect on asthma control [47]. Meanwhile, it was ascertained that uLTE4 may be useful as a potent biomarker of atopic, viral or IgE-mediated asthma in children [48,49]. It was shown that uLTE4 is also an effective biomarker to determine aspirin intolerance in subjects with asthma [50], and of exposure to atopic and non-atopic asthma triggers, recent exacerbations, and early development of childhood atopy. It is also used for predicting response initiation or step-up therapy with LTRAs [51].

### 2.4. Estimated Glomerular Filtration Rate and Kidney Function

PTS and SHS exposure are known risk factors for kidney disease [52,53]. In adolescents, Omoloja et al. [54] described SHS exposure not only as a risk factor for chronic kidney disease (CKD) but also as a risk factor independently or together with CKD for adverse cardiovascular outcomes. The estimated glomerular filtration rate (eGFR) as a marker for kidney function was decreased in adolescents exposed to tobacco smoke including SHS and, thus, may affect kidney function in early life [55]. In children with CKD, an association between SHS exposure and nephrotic range proteinuria was found [56]. PTS exposure may lead to lower eGFR and smaller kidney volume in children of school age [57].

### 2.5. Cardiovascular Status

SHS is a risk factor for coronary heart disease [58]. SHS can lead to endothelial dysfunction, a main factor for cardiovascular disease [59]. Cardiovascular diseases are also caused by PTS exposure [60]. Groner et al. [61] found in 9 to 18 years old children and adolescents that SHS exposure is associated with high levels of soluble intercellular adhesion molecule 1 (s-ICAM1, transmembrane protein released at atherosclerotic lesions) and negatively associated with the prevalence of endothelial progenitor cells. That might lead to both vascular endothelial stress and lower capability to vascular repair. Enlarged carotid artery intima-media thickness (IMT) can be induced by SHS exposure in early life and is a risk factor of atherosclerosis in adulthood [62]. In neonates, it was found that the aortic IMT increased due to PTS exposure [63]. Five-year-old children exposed to PTS had greater carotid IMT than non-exposed [64]. It was found in adults exposed to SHS in childhood that they had greater carotid IMT [65]. In a recent study, it was shown that SHS exposure in childhood leads to an increased risk of atrial fibrillation in adulthood [66].

The described literature shows that exposure to PTS and SHS has negative effects on vascular walls in early life and is harmful to the cardiovascular system also in later life.

### 2.6. C-Reactive Protein

C-reactive protein (CRP) is involved in the systematic inflammation response. On the other side, CRP is a risk factor for adiposity in children and for the development of atherosclerosis [67,68]. It was shown in non-smoking youths that SHS exposure is associated with a significant increase in CRP serum concentration [69]. Similar results were found in an exploration of 10-year-old children exposed to SHS [70]. High-sensitivity CRP (hsCRP) values in serum were increased in three- to five-year-old SHS-exposed children in a dose-response manner [71]. Additionally, SHS exposure in early life could increase the risk of increased hsCRP levels in adulthood [72].

## 3. Immune Status

Regulatory T-cells (Tregs) are essentially involved in the immune regulation of atopic diseases [73]. Hinz and colleagues [74] found in a prospective birth cohort study lower Treg cell numbers in cord blood of children exposed to PTS. They concluded this might be a predictor for early atopic dermatitis or sensitization to food allergens in early life. It was shown that maternal smoking during pregnancy leads to a higher expression of microRNA-223 (miR-223) both in maternal blood cells and in cord blood cells. This was, in turn, associated with lower Treg cell numbers [75]. The underlying cause could be the regulative effect of miR-223 on the transcription factor FoxO1 that plays in turn significant roles in T-cell regulation [76,77]. A combination of lower Treg cell numbers and higher numbers of T-helper type 17 (Th17) cells were ascertained in children exposed to SHS [78]. Th17/Treg imbalance is a key component of asthma severity [79]. In SHS-exposed healthy adolescents, a decrease in circulating CD3+ and CD4+ memory cells accompanied by an increase in circulating naïve CD3+ and CD4+ T-cell subsets and absolute CD4+CD45RA+ cell counts in a dose-effect response was found [80]. Whereby, this systemic immunological response could be a possible initiator for diseases in later life. The proportion of IFN-γ producing CD8+ cells was lower in adenoids from SHS-exposed children, possibly resulting in an increasing predisposition to respiratory infections [81]. In an in vitro investigation, it was ascertained that adenoidal B-lymphocytes of SHS exposed and/or atopic children produce more IgA and IgM. For this purpose, B-lymphocytes of ectomised tonsils of children with adenoidal hypertrophy were cultured and stimulated [82].

Yao et al. [83] showed in an investigation of immunoglobulin E (IgE) levels against 40 allergens that SHS exposure of children and adolescents is significantly associated with IgE sensitization to grass pollen, cockroaches, and certain foods (cow’s milk, egg white, crab, shrimp, codfish, soybean, potato, peanut, almond, garlic, and cheese), but not with sensitization against mites, mould, and latex. SHS exposure in early infancy, but not during pregnancy, was associated with an increased risk of food sensitization (IgE antibody reactivity against cow’s milk, egg white, soybean, peanut, cod, and wheat) up to age 16 years [84]. In general, SHS exposure in early childhood increases the risk of allergic sensitization [85].

Kopp et al. [28] showed in infants and young children with cystic fibrosis (CF) disproportionately exposed to SHS that inflammatory gene expression and arachidonic acid (AA) metabolism are altered, resulting in an impaired bacterial clearance. Among others, they also reported an association between SHS exposure and decrease in the AA metabolite prostaglandin D_2_, suppression of two prostaglandin genes (prostaglandin reductase 2, PTGR2 and prostaglandin E synthase 3, PTGES3), and overexpression of regulatory factor X2 gene.

## 4. Lipid Profile

It was described that lower serum levels of high-density lipoprotein-cholesterol (HDL-C) in children are associated with SHS exposure, suggesting an increased risk of arteriosclerosis [86]. SHS-exposed children showed significantly higher values of triglycerides, total cholesterol and low-density lipoprotein-cholesterol (LDL-C) in combination with lower values of HDL-C, and enhanced peripheral lymphocyte apoptosis possibly caused by the altered lipid profile [87]. In schoolchildren, an association between SHS exposure, higher values of triglycerides and lower HDL-C levels was reported, meaning an enhanced risk of obesity and metabolic syndrome [88]. HDL-C reductions were also found in SHS-exposed toddlers [89] and in healthy 8-year-old children exposed to PTS [90]. Lower HDL-C levels were also observed in female adolescents, but not in male ones [91]. Another study showed an increase in apolipoprotein (Apo) B and ratio ApoB/ApoA-1 in adolescents exposed to SHS [92]. Accordingly, low ApoA-1 and high lipoprotein-associated phospholipase A2 concentrations were noticed at 10-year-old children exposed to SHS [70]. High triglycerides and low HDL-C levels were found in women 18–44 years after PTS exposure [93]. Regarding lipid metabolism of neonates, several phosphatidylcholine (lipid metabolites) levels were affected by PTS exposure and differed significantly in sera probes from mothers and cord blood [94].

These study results report that PTS and SHS exposure has a negative influence on the lipid profile in children up to adulthood accompanied by associated health effects. In contrast, Zakhar and colleagues [95] reported no material association between SHS exposure and lipid profiles in children. However, the authors concluded that an effect on abnormalities of serum lipid levels might require a longer tobacco smoke exposition time.

## 5. Oxidative Stress

Oxidative stress (OS) is characterised by increased intracellular levels of reactive oxygen species (ROS) [96]. In severe cases, OS can lead to cell and tissue injury and even cell death [97], and is associated, among others, with asthma [98] and cardiovascular events in adulthood [99]. OS can lead to many foetal and neonate diseases because free radicals, with their harmful effects like cellular, tissue and organ damage, cannot be fought by a weakened antioxidant system [100]. OS plays a key role in the pathogenesis of COPD [11]. SHS is an important inductor of OS, particularly among adolescents and children [101,102].

The enzyme nicotinamide adenine dinucleotide phosphate oxidase-2 (Nox2) forms ROS by generating super-oxide [103]. It was found that Nox2 activity was higher in children exposed to SHS, leading to an increase in OS and to artery dilation [104]. OS may play a major role in the metabolic syndrome, too [105]. It is described that SHS leads to metabolic syndrome in adolescents [106] and children, especially in combination with low intake of vitamin E or omega-3 polyunsaturated fatty acids [107]. Neonates exposed to PTS were shown to have lower levels of adiponectin and higher levels of visfatin, indicating a less beneficial OS profile compared to non-exposed new-borns [108]. In an urban study, adolescents exposed to SHS showed an increase in urinary 15-F_2t_-isoprostane, a specific product of lipid peroxidation and biomarker for OS level [109].

Kobayashi et al. [110] demonstrated in their study of 2014 that SHS-induced OS reduces the histone deacetylase-2 (HDAC2) function by the activation of phosphoinositide-3-kinase (PI3K), which in turn reduces corticosteroid sensitivity. This corticosteroid insensitiveness make treatment by corticosteroids of severe asthma in children even more difficult [111]. In 2010, Cohen et al. [112] reported that PTS exposure reduces the positive effect of inhaled corticosteroids in asthmatic children. Thus, asthmatic children need higher doses of inhaled corticosteroids for treatment [113].

## 6. Hormone Status

Several investigations have suggested that PTS exposure leads to hormonal changes in childhood and later life, with increasing risks for adiposity and metabolic and endocrine dysfunction [114]. A higher plasma concentration of the appetite-stimulating hormone ghrelin was shown in 19-year-old young adults exposed to PTS [115]. PTS exposure can influence the foetal endocrine hormone system, like changes in the production of cortisol, oestrogens, androgens, and hormones of the pituitary [116]. PTS and SHS exposure can alter reproductive hormones like luteinizing hormone and inhibin B and, therefore, influence the puberty of girls [117]. Another study showed that daughters exposed to PTS had an earlier age of menarche, but only non-significant lower levels of testosterone and dehydroepiandrosterone-sulphate, and no changes in serum levels of other reproductive hormones [118]. It was found that the positive association between high serum thyrotropin (TSH) levels and body mass index is stronger in SHS-exposed adolescents aged 11–17 than in the unexposed [119]. This boosting effect of SHS exposure was not found in children aged 3–11. Filis et al. [120] reported an association between disturbances of foetal thyroid gland development and maternal smoking and maternal overweight, possibly resulting in influences on endocrine function, foetal metabolism, brain development, cardiac output and adverse post-natal health effects. The hormone leptin will be expressed in adipocytes, regulates food intake and basal metabolism, and correlates with the body mass index with influence on vascular function [121]. In 10-year-old children, SHS exposure was shown to increase leptin plasma concentration [70].

## 7. Genetic Predisposition

The genetic predisposition may influence the health effects of PTS and SHS exposure in childhood until later life. In the following, different genetic variants of several genes, e.g., glutathione S-transferase genes, interleukin (IL) genes or variants at chromosome 17, are described which may lead to increased adverse health effects (e.g., asthma, wheezing, sudden infant death syndrome, congenital heart defects). Table 2 shows a summary and additional information to the presented genetic predispositions.

### 7.1. Glutathione S-Transferase (GST) Genes

GSTs, including the isoforms GSTP1, GSTM1, and GSTT1, are multifunctional enzymes for cellular detoxification and are involved in oxidative stress pathways, among others, in the lung [122], and are associated, among others, with asthma and wheezing in children exposed to PTS or SHS [17,18]. In a Swedish prospective birth cohort study (n = 982 wheezers and non-wheezers up to age 4), children with specific variations of three single nucleotide polymorphisms (SNPs) in the GSTP1 gene (Ile105Val, Intron 5, Intron 6) and exposure to SHS had a higher risk of early childhood wheezing, but with no allergic sensitization [123]. In Taiwanese school children (216 wheezers and 185 non-wheezers), homozygosity for GSTP1 Val-105 was significantly associated with both current and ever wheezing, whereas homozygosity for GSTP1 Ile-105 was associated with current wheezing but not with ever wheezing [124]. In a study at 504 children and adolescents with asthma aged between 3 and 21 years, children exposed to SHS and with the GSTP1 polymorphism GG (Val105Val) at nucleotide 1695 or null for the GSTM1 gene were more prone to asthma [125]. A Taiwanese longitudinal birth cohort study (n = 591 children, 138 asthmatics at age 6) found that the GSTM1 null genotype could have a gender-specific effect on the development of asthma. Girls who were not exposed to PTS were more protected against asthma. The GSTM1 null type becomes a risk factor for prenatal exposed boys and girls [126]. Another study in 1124 schoolchildren aged 7–12 years described that SHS-exposed children with the GSTP1 polymorphism AA (Ile105Ile) at nucleotide 1695 showed an increased risk of asthma, too. This effect was boosted by a low intake of vitamin A [127]. At 1132 school children, a common haplotype of GSTP1 (including the polymorphism Ile105Val) was associated with a lower risk of respiratory illness. This effect of protection was lost in those children exposed to PTS or SHS [128].

In contrary to these findings, Turner et al. [129] found no evidence for a significant association between the investigated GST variants GSTP1 Val-105 (n = 3692 children), GSTM1 null and GSTT1 null (n = 2362 children), SHS exposure and asthma attacks in children. The authors concluded that the findings of previous studies, positive associations between GSTT1 null and asthma and GSTM1 null and asthma severity, were false-positive findings.

GST genes may not only be associated with wheezing and asthma. PTS exposure among children with the GSTM1 null genotype was also associated with increased adverse effects on cognition in pre-schoolers [130]. Regarding sudden infant death syndrome (SIDS), a significant association was found between tobacco smoke exposure and GSTM1 null genotype, but no association between GSTT1 null and SIDS [131]. Taiwanese children carrying GSTM1 null and homozygous GSTP1 Ile-105 showed an increasing prevalence of atopic dermatitis when exposed to PTS [132]. Li et al. [133] found an association between congenital heart defects (CHDs) and polymorphisms in GST genes in neonates induced by maternal smoking. Regarding the lung function in adolescents, an atopy cohort study found that exposed children may be more susceptible to lung function impairment in later life if they are homozygous for GSTP1 Ile-105 allele or carriers of the null mutation at GSTM1 or GSTT1 [134].

### 7.2. Anti-Inflammatory Cytokine Genes

Anti-inflammatory cytokines like tumour necrosis factor-alpha (TNF-α), transforming growth factor-beta (TGF-β) and interleukins (IL) regulate the human immune response by controlling the pro-inflammatory response [30]. In this respect, children with the TNF-308A variant (both homozygous and heterozygous) were more susceptible to respiratory illness by SHS compared to children homozygous for the common TNF-308G allele [135]. SNP variations of TNF (-857C/T, Intron 1, Intron 3) in combination with early maternal smoking interacted with a higher risk of early childhood wheezing but not with allergic sensitization [123].

Regarding the TGF-beta1 gene, it was found that the combination of PTS exposure and the TGF-beta1-509TT genotype of the child increased the risk of childhood asthma [136].

IL-4, IL-5 and IL-13 are associated with asthma, too [31]. It was found in African-American infants exposed to SHS that the CT and TT genotypes for IL-4 C-589T increase the risk of wheezing [137]. In an investigation of the Isle of Wight birth cohort, the common IL 13 haplotype pair CCG/CCG and a single SNP increased the negative effect of maternal smoking during pregnancy on early-onset persistent wheezing and persistent asthma in childhood [138].

### 7.3. CD14 Gene

CD14 is a receptor for lipopolysaccharide (LPS, endotoxin) on the surface of macrophages, monocytes, and neutrophil blood cells (membrane-anchored = mCD14). CD 14 exists also as a soluble serum protein (sCD14). Both are important molecules regarding endotoxin-dependent signal transduction [139]. It was reported for SHS-exposed children aged four years that an AA genotype in the 3’untranslated region of CD14 was associated with lower IgE levels than in children not exposed. No differences in IgE levels in exposed and non-exposed children were found for the genotype CC/CA [140]. Hussein et al. [141] found that the CD14 genotypes -159TT and -550TT are associated with elevated serum IgE levels in Egyptian children exposed to SHS and may contribute to atopy predisposition.

### 7.4. Variants at Chromosome 17 Region q21

Bouzigon et al. [142] tested 36 SNPs at chromosome 17q21 for an association with early-onset asthma. They found 11 genetic variants increasing the risk of early-onset asthma, especially at early-life exposure to SHS. These 11 SNPs distributes to four genes: IKZF3 (involved in lymphocyte development), ZPBP2 (zona pellucida-binding protein 2), GSDMB (encodes one gasdermin protein which is involved in skin differentiation and epithelial barrier function) and ORMDL3 (encodes a transmembrane protein). The authors discussed that GSDMB and ORMDL3 in particular may be involved in viral respiratory infections, leading in turn to an increased risk of asthma. The interaction among 17q21variants, SHS exposure of children, and paediatric asthma was confirmed in North Americans of European ancestry, but without the finding of age-of-onset [143]. Both PTS exposure and SHS exposure in early postnatal life and a 17q21 SNP variant increased the asthma-like symptoms in preschoolers [144]. For one SNP on 17q21, an influence on childhood asthma was assumed, but not on asthma risk in adulthood [145]. No association was found for 17q21 variants and late-onset asthma [146].

### 7.5. ATPase-Related Genes and Bronchial Hyper-Responsiveness

A genetic variant in the DNAH9 gene (chromosome 17p11) in combination with early SHS exposure seems to be linked with bronchial hyper-responsiveness (BHR), an important characteristic for asthma [147,148]. Another two candidate genes, ATP8A1 (chromosome 4) and ABCA1 (chromosome 9), interacting with early-life SHS exposure and BHR, were identified [149].

### 7.6. Mannose-Binding Lectin-2 (MBL2) Gene

Several genetic polymorphisms and haplotypes in the MBL2 gene increased the lung cancer risk in adults exposed to SHS in their childhood [150]. This is due to the genetic background of MBL2 with consequences in inherent immunity.

### 7.7. Flavin-Monooxygenase-3 (FMO3) Gene

The common polymorphism G472A of the FMO3 gene may be a risk factor for SIDS in children exposed to PTS [151]. The authors declared their study as the first one demonstrating an interaction between a gene and an environmental factor in SIDS.

### 7.8. O-Sialoglycoprotein Endopeptidase (OSGEP) Gene

CHDs are among the most prevalent birth defects and are a major cause of death in early childhood [152]. In infants, four SNPs in the OSGEP gene were described as increasing the risk moderately for the occurrence of CHDs in the presence of maternal smoking [153].

### 7.9. MSX1 Gene

MSX1, a homoeobox gene playing key roles in craniofacial development, is involved in the formation of orofacial clefts (OFC), a common birth defect [154]. The combination of allele 4 homozygosity of the child and PTS exposure increased the risk of OFC [155].

## 8. Protein Expression in Foetal Liver

In a study on liver samples from aborted foetuses, maternal smoking was gender-specifically associated with changes in the expression pattern of metabolic enzyme transcripts [157]. The authors detected in male foetuses exposed to maternal smoke an expression increase of eight mRNA transcripts (GSTT1, GSTP1, CYP1A1, EPHX1, NQO1, AHR, AS3MT, GLRX2) and a decrease of three transcripts (GGT1, CYP2R1, CAR). In female foetuses, an increase of two transcripts (CYP3A7, EPHX1) was detected. They emphasised the impact on foetal liver activity by environmental toxicants like tobacco smoke. Drake and colleagues [158] showed in foetal livers widespread sex-specific effects by maternal smoking on the expression of key enzymes of the 1-carbon metabolism, level of vitamin B12 and homocysteine in foetal plasma.

Filis et al. [159] described a broad range of dysregulated protein expression in foetal livers induced by maternal smoking in a sex-specific manner, too. Among others, they found expression differences in proteins related to detoxification, homeostasis, protein processing and secretion, necrosis and cancer development, proliferation, apoptosis, and inflammation.

## 9. Dysregulation of Diverse MicroRNAs

MicroRNAs (miRNAs) are small RNAs with about 22 nucleotides which regulate posttranscriptional gene expression [160]. Placental miRNAs are co-responsible for the development of maternal placenta and foetus. It was ascertained that smoking during pregnancy can down-regulate miRNA-16, miRNA-21, and miRNA-146a in the placenta [161]. On the other side, an up-regulation of miRNA-223 in maternal and cord blood by PTS exposure was noticed to be associated with lower Treg cell numbers in cord blood and succeeding risks for allergies [75]. Lower expression of miR-199a1 in children induced by PTS exposure was reported [162]. This down-regulation leads to lower expression of the receptor-tyrosine-kinase AXL gene and might increase the risks of bronchitic symptoms, especially in combination with higher AXL methylation [162,163]. A recent study showed positive associations between indoor air pollution, e.g., induced by SHS, higher serum levels of miR-155 and asthma in childhood [164].

## 10. Leukocyte Telomere Length

Telomeres are repetitive DNA sequences. Every cell divide shortens the telomeres. The length of telomeres is associated with the biological age: the shorter the telomere, the higher the biological age [165]. A recent birth cohort study (n = 1396 children aged 5 to 12 years) suggested for the first time that PTS exposure in early life can shorten leukocyte telomeres in children, inducing premature biological ageing, even at an early age [166].

## 11. DNA Methylation

Tobacco smoke is a potent environmental factor for DNA methylation [167]. PTS exposure can induce epigenetic changes in the foetus with sequelae in later life, among others altering risks for allergic diseases, metabolic diseases, or cardiac disorders [168,169,170]. Many studies focussed on DNA methylations with differences in the global pattern: hyper-methylation in placental tissue and hypo-methylation in cord blood and several types of foetal cell or tissue [171]. Different methylation patterns were found for different placental and foetal genes [74,172,173,174,175,176,177]. Joubert et al. [178] reported in an epigenome-wide analysis of >470,000 individual cytosine-guanine dinucleotide (CpG) sites in cord blood significant methylation changes at 26 CpG sites in 10 genes associated with maternal smoking. That confirmed and extended a study by Markunas and colleagues [179]. The results suggest that changes in DNA methylation triggered by smoking during pregnancy might underlie some consequences of maternal smoking on offspring (e.g., regarding processes at placental and embryonic development).

A recent review article by Zakarya and colleagues presented the epigenetic influences of maternal smoking and the resulting poor effects on the respiratory system of offspring [180]. Den Dekker et al. found 59 differentially methylated regions in the DNA of cord blood altering childhood lung function, some of them additionally associated with childhood asthma or adult COPD [181]. It should also be mentioned that CpG methylation in the gene loci of Forkhead box P3 (FOXP3) and interferon gamma-γ (IFNγ) can be accelerated by SHS exposure [182]. In turn, this can affect the function of T-cells and is associated with asthma. PTS exposure has also been related to lower promoter methylation in the neuropeptide S receptor 1 (NPSR1), significantly associated with asthma [183], and higher AXL gene methylation at birth, increasing bronchitic symptom risk in childhood [162].

In foetal livers, alterations to the methyl donor availability of vitamin B12 were described [158]. This might lead to changes in DNA methylation of the genes IGF2 and NR3C1 (glucocorticoid receptor) induced by maternal smoking with possible consequences on foetal growth and later cardio-metabolic or neuropsychiatric disorders [158].

Finally, it should not go unmentioned that both opioid receptor mu-1 gene (OPRM1) and PTS exposure are factors for the preference for fatty foods [184,185]. It was assumed that PTS exposure is able to epigenetically modify an OPRM1 allele (rs2281617) increasing fat preference [186]. These findings are potential reasons for the described risk of obesity in offspring exposed to PTS [187].

## 12. Conclusions

Worldwide, many people are still exposed to SHS, especially in public places, at home or in vehicles. Many children will be affected in health by tobacco smoke exposure both before and after birth. The adverse health effects range from prenatal epigenetic changes, triggering of diseases in childhood to health detriment in later life.

This review summarised scientific knowledge of molecular and genetic mechanisms in children induced by PTS and SHS exposure. Tobacco smoke can alter those mechanisms negatively and can cause several diseases. PTS and SHS increase children’s cancer risk, trigger or worsen allergies and asthma in children, and are harmful to their respiratory tract and cardiovascular system. Genetic predisposition may worsen the health effects of exposure. PTS can lead to poor birth outcomes, pregnancy complications, and foetal maldevelopment. It became clear that further research on mechanisms underlying tobacco smoke-induced diseases is necessary, partly because of contradictory study results. Transnational biomonitoring can show the exposition burden by tobacco smoke, especially in children. In this context, the Consortium to Perform Human Biomonitoring on a European Scale (COPHES), funded by the European Union’s Seventh Framework Programme [188,189], and DiMoPEx (Diagnosis, Monitoring and Prevention of Exposure-related Non-Communicable Diseases) by the European Cooperation in Science and Technology [190,191] should be mentioned.

A deeper understanding of health damage by tobacco smoke exposure, which especially affects the child’s developing body, helps the public and policymakers to react adequately with public health interventions, tightening of smoke-free policies, e.g., smoking bans in vehicles with children, health communication campaigns, and behavioural interventions. It is important to emphasise that children in particular should be protected from exposure to tobacco smoke.

## Figures and Tables

**Table 1:** Summary of changes in children induced by second-hand smoke (SHS) and prenatal tobacco smoke (PTS) exposure.

Topic	Effects on Children	Test Material	Source	Association
**Biomarkers**				
Matrix metalloproteinase-9 (MMP-9)	MMP-9 increased	Nasal secretion	SHS	Allergy, asthma, chronic bronchitis [26], no effect [29]
Cytokine Interleukin (IL)	IL-1β increased	Saliva	SHS	Inflammatory processes [35,36]
	IL-1β decreased	Blood	SHS	Inflammatory processes [34]
	IL-4 decreased	Blood	SHS	Inflammatory processes [34]
	IL-5 decreased	Blood	SHS	Inflammatory processes [34]
	IL-6 increased	Saliva	SHS	Inflammatory processes [36]
	IL-8 increased	New-born dried blood	PTS	Inflammatory processes [33]
	IL-8	Nasal secretion	SHS	no effect by SHS [29]
	IL-8	Saliva	SHS	no effect by SHS [36]
	IL-13 increased	Airway secretion	SHS	Inflammatory processes [37]
	IL-17	Nasal secretion	SHS	no effect by SHS [29]
Cytokine Interferon gamma (IFN-γ)	IFN-γ decreased	Blood	SHS	Inflammatory processes [34]
Tumour necrosis factor alpha (TNF-α)	TNF-α increased	Saliva	SHS	Inflammatory processes [36]
Urinary leukotriene E4 (uLTE4)	uLTE4 increased	Urine	SHS	Asthma [42,43]
Estimated glomerular filtration rate (eGFR)	eGFR decreased	Serum	SHS	Kidney function, proteinuria [55,56]
Intercellular adhesion molecule 1 (s-ICAM1)	s-ICAM1 increased	Serum	SHS	Endothelial stress [61]
Intima-media thickness (IMT)	IMT increased	Ultrasonography	SHS, PTS	Atherosclerosis [64,65]
C-reactive protein (CRP)	CRP increased	Serum	SHS	Inflammation response [69,71,72]
**Immune status**				
Regulatory T-cells (Tregs)	Treg cell number decreased	Cord blood, blood	PTS, SHS	Atopy, asthma [74,75,78]
T-helper 17 (Th17) cells	Th17 cell number increased	Blood	SHS	Asthma severity [78]
T-cells subsets	Circulating CD3+ and CD4+ memory T-cell number decreased	Blood	SHS	Systemic immunological response [80]
	Circulating CD3+ and CD4+ naïve T-cell number increased	Blood	SHS	Systemic immunological response [80]
	CD4+CD45RA+ T-cell number increased	Blood	SHS	Systemic immunological response [80]
	CD8+ T-cell number decreased	Adenoids	SHS	Systemic immunological response [81]
Immunoglobulins A and M (IgA, IgM)	IgA and IgM increased	Adenoids	SHS	Systemic immunological response [82]
Immunoglobulin E (IgE)	Immune response to allergens increased	Serum	SHS	Allergy [83,84,85]
**Lipid profile**				
High-density lipoprotein-cholesterol (HDL-C)	HDL-C decreased	Blood	SHS, PTS	Arteriosclerosis, obesity, metabolic syndrome [86,87,88,89,90,91,93], no effect by SHS [95]
Low-density lipoprotein-cholesterol (LDL-C)	LDL-C increased	Blood	SHS, PTS	Arteriosclerosis, obesity, metabolic syndrome [87], no effect by SHS [95]
Triglycerides	Triglycerides increased	Blood	SHS, PTS	Arteriosclerosis, obesity, metabolic syndrome [87,88,93], no effect by SHS [95]
Apolipoprotein A-1 (ApoA-1)	ApoA-1 decreased	Blood	SHS	Arteriosclerosis, obesity, metabolic syndrome [70]
Apolipoprotein B (ApoB)	ApoB increased	Blood	SHS	Arteriosclerosis, obesity, metabolic syndrome [92]
**Oxidative stress (OS) increased**				In general: cell, tissue and organ injury, cell death; asthma, COPD, cardiovascular events, metabolic syndrome [100,101,102,107]
Nicotinamide adenine dinucleotide phosphate oxidase-2 (Nox2)	Nox2 increased	Serum	SHS	Artery dilation [104]
Adiponectin	Adiponectin decreased	Cord blood	PTS	Lipid peroxidation increased [108]
Pre-B-cell colony enhancing factor (Visfatin)	Visfatin increased	Cord blood	PTS	Lipid peroxidation increased [108]
Urinary 15-F_2t_-isoprostane	Urinary 15-F_2t_-isoprostane increased	Urine	SHS	Lower lung function parameters [109]
Histone deacetylase-2 (HDAC2)	HDAC2 decreased	Broncho-alveolar lavage fluid	SHS	Corticosteroid-insensitiveness leads to impairment of severe asthma treatment [110]
**Hormonal changes**				In general: Metabolic and endocrine dysfunction (foetal, in childhood, and later life) [114,116]
Ghrelin	Ghrelin increased until early adulthood by PTS exposure	Plasma	PTS	Metabolic disorders [115]
Leptin	Leptin increased	Plasma	SHS	Impairing of vascular function, BMI [70]
Adiponectin	Adiponectin decreased	Cord blood	PTS	OS increased, lipid peroxidation increased [108]
Luteinizing hormone (LH)	In girls, LH decreased by PTS exposure but increased by current SHS exposure	Blood	PTS, SHS	Reproductive development [117]
Inhibin B (InB)	In girls, InB decreased by PTS exposure with no effect by current SHS exposure	Blood	PTS, SHS	Reproductive development [117]
Thyrotropin (TSH)	TSH decreased	Serum	SHS	Hypothyroidism, BMI [119]
Foetal triiodothyronine (T3), thyroxine (T4) and TSH	T3, T4 and TSH decreased (possibly by downregulation of foetal thyroid transcripts GATA6 and NKX2-1)	Foetal plasma	PTS	Disorder of foetal thyroid development and endocrine function [120]
Foetal corticotropin-releasing hormone (CRH), adrenocorticotrophin (ACTH), cortisol, gonadotropins, androgens, oestrogens	Changes in foetal steroidogenesis	Foetal plasma	PTS	Multiple pathophysiological effects (foetal and later in life) by endocrine dysfunction [116]

**Table 2:** Summary of addressed single nucleotide polymorphisms (SNPs) regarding genetic predisposition to tobacco smoke susceptibility.

Gene (Chromosome)	SNP	Risk Allele	Association
GSTP1 Exon 5 (11q13)	rs1695 (Val-105 or Ile105Val)	AG (Ile105Val)	Early childhood wheezing [123]; protection against respiratory illness was lost by PTS exposure [128]; no effect in asthma [129]
		GG (Val105Val)	Asthma [125]; no effect in asthma [129]; current and ever wheezing [124]
		AA (Ile105Ile)	+ low vitamin A intake: asthma [127]; no effect in asthma [129]; current wheezing [124]; atopic dermatitis [132]; lung function impairment in later life [134]
GSTP1 Intron 5 (11q13)	rs749174	TT	Early childhood wheezing [123]
GSTP1 Intron 6 (11q13)	rs1871042	TT	Early childhood wheezing [123]
TNF Promoter (6p21)	rs1800629 (-308)	AA/AG	Respiratory illness [135]
TNF Promoter (6p21)	rs1799724 (T-857C)	CC	Early childhood wheezing [123]
TNF Intron 1 (6p21)	rs1800610	CC	Early childhood wheezing [123]
TNF Intron 3 (6p21)	rs3093664	AG/GG	Early childhood wheezing [123]
TGFB1 Promoter (19q13)	rs4803457 (C-509T)	TT	Asthma [136]
IL-4 (5q31)	rs2243250 (C-589T)	TT/CT	Wheezing [137]
IL-13 Exon 4 (5q31)	rs20541 (G/A)	GG	Early onset persistent wheeze and persistent asthma [138]
IL-13 haplotype pair (Promoter, Intron 1, Exon 4) (5q31)	rs1800925 (C/T), rs2066960 (C/A), rs20541 (G/A)	CCG/CCG	Early onset persistent wheeze and persistent asthma [138]
CD14 (5q31)	3’untranslated region (UTR)	AA	Lower IgE levels [140]
CD14 Promoter (5q31)	rs2569190 (C-159T)	TT	Elevated IgE levels, atopy [141]
CD14 (5q31)	C-550T	TT	Elevated IgE levels, atopy [141]
IKZF3 Intron 3 (17q21)	rs9303277	C	Increased risk of early-onset asthma enhanced by SHS [142]; confirmed in Caucasians without age of onset [143]
ZPBP2 Exon 2 (17q21)	rs11557467 (I151S)	G	Increased risk of early-onset asthma enhanced by SHS [142]; confirmed in Caucasians without age of onset [143]
GSDMB Exon 8 (17q21)	rs2305480 (P298S)	G	Increased risk of early-onset asthma enhanced by SHS [142]; confirmed in Caucasians without age of onset [143]; asthma-like symptoms [144]
GSDMB Exon 8 (17q21)	rs2305479 (G291R)	C	Increased risk of early-onset asthma enhanced by SHS [142]
GSDMB Intron (17q21)	rs4795400	C	Increased risk of early-onset asthma enhanced by SHS [142]
GSDMB Intron (17q21)	rs9303281	A	Increased risk of early-onset asthma enhanced by SHS [142]
GSDMB Intron 1 (17q21)	rs7219923	T	Increased risk of early-onset asthma enhanced by SHS [142]
GSDMB Intron 2 (17q21)	rs2290400	C	Increased risk of asthma in Caucasians enhanced by SHS [143]
GSDMB Intron 2 (17q21)	rs7216389	T	Increased risk of asthma in Caucasians enhanced by SHS [143,145]
GSDMA Exon 2 (17q21)	rs3894194	A	Increased risk of asthma in Caucasians enhanced by SHS [143]
GSDMA Intron 6 (17q21)	rs3859192	?	Increased risk of asthma in Caucasians enhanced by SHS [143]
ORMDL3 Intron (17q21)	rs8076131	A	Increased risk of early-onset asthma enhanced by SHS [142]
LRRC3C Intron (17q21)	rs8079416	?	Increased risk of asthma in Caucasians enhanced by SHS [143]
Intergenic region (17q21)	rs8069176	G	Increased risk of early-onset asthma enhanced by SHS [142]
Intergenic region (17q21)	rs4795405	C	Increased risk of early-onset asthma enhanced by SHS [142]; confirmed in Caucasians without age of onset [143]
Intergenic region (17q21)	rs4794820	G	Increased risk of early-onset asthma enhanced by SHS [142]
Intergenic region (17q21)	rs8067378	?	Increased risk of asthma in Caucasians enhanced by SHS [143]
DNAH9 Intron (17p11)	rs7225157	?	Bronchial hyperresponsiveness [148]
ATP8A1 Intron (4p13)	rs17448506	?	Bronchial hyperresponsiveness [149]
ABCA1 Intron (9q31)	rs2253304	?	Bronchial hyperresponsiveness [149]
MBL2 (10q21)	rs5030737	AA	Increased risk of lung cancer in later life [150]
MBL2 Intron (10q21)	rs1838066	CC	Increased risk of lung cancer in later life [150]
MBL2 Intron (10q21)	rs7095891	TT	Increased risk of lung cancer in later life [150]
MBL2 (10q21)	rs2165810	TT	Increased risk of lung cancer in later life [150]
FMO3 (1q24)	rs2266782 (G472A)	AA	Risk factor for sudden infant death syndrome [151]
OSGEP Intron (14q11)	rs1320150	AG	Increased risk of congenital heart defects [153]
OSGEP Intron (14q11)	rs938881	?	Increased risk of congenital heart defects [153]
OSGEP (14q11)	rs2275007	?	Increased risk of congenital heart defects [153]
OSGEP Intron (14q11)	rs883037	?	Increased risk of congenital heart defects [153]
MSX1 Intron allele 4 (4p16)		Homozygosity of 9 repeats of the A4 CA marker	Increased risk of nonsyndromic orofacial clefts [155]

Additional information was given from the National Center for Biotechnology Information (NCBI) of the U.S. National Library of Medicine database [156]. The table presents risk alleles associated with a disease. ? = risk allele not reported. SNP = single nucleotide polymorphism. A = adenine. C = cytosine. G = guanine. T = thymine.
